# Transcriptome profiles of the skeletal muscle of mature cows during feed restriction and realimentation

**DOI:** 10.1186/s13104-021-05757-8

**Published:** 2021-09-16

**Authors:** Hannah C. Cunningham-Hollinger, Larry A. Kuehn, Kristi M. Cammack, Kristin E. Hales, William T. Oliver, Matthew S. Crouse, Celine Chen, Harvey C. Freetly, Amanda K. Lindholm-Perry

**Affiliations:** 1 University of Wyoming, Laramie, WY 82071 USA; 2 South Dakota State University, West River Ag Center, Rapid City, SD 57702 USA; 3 USDA, ARS, U.S. Meat Animal Research Center, P.O. Box 166, Clay Center, NE 68933 USA; 4 USDA, ARS, Beltsville Human Nutrition Research Center, Beltsville, MD 20705 USA

**Keywords:** Skeletal muscle, Microarray, Transcriptome, Body weight gain

## Abstract

**Objective:**

Realimentation can compensate for weight loss from poor-quality feedstuffs or drought. Mature cows fluctuate in body weight throughout the year due to nutrient availability. The objective of this study was to determine whether cows that differ in weight gain during realimentation also differ in the abundance of transcripts for enzymes associated with energy utilization in skeletal muscle. Mature cows were subjected to feed restriction followed by ad libitum feed. Skeletal muscle transcriptome expression differences during the two feeding periods were determined from cows with greater (n = 6) and less (n = 6) weight gain during the ad libitum feeding period.

**Results:**

A total of 567 differentially expressed genes (408 up- and 159 down-regulated) were identified for the comparison of restriction and ad libitum periods (P_Bonferroni_ < 0.05). These genes were over-represented in lysosome, aminoacyl-tRNA biosynthesis, and glutathione metabolism pathways. Validation of the expression of five of the genes was performed and four were confirmed. These data suggest that realimentation weight gain for all cows is partially controlled by protein turnover, but oxidative stress and cellular signaling pathways are also involved in the muscle tissue. This dataset provides insight into molecular mechanisms utilized by mature cows during realimentation after a period of low abundance feed.

**Supplementary Information:**

The online version contains supplementary material available at 10.1186/s13104-021-05757-8.

## Introduction

Cattle producers are faced with the challenge of increasing production to feed the growing population, and over 2/3 of the budget is the feed to achieve production goals. Compensatory gain (CG) increases production while maintaining or even decreasing inputs and affects an animal’s lean tissue and fat deposition. Forage quality fluctuates with season; thus, the mature beef cow can experience periods of nutrient restriction followed by realimentation. This, along with the utilization of CG for improved feed efficiency, highlights the necessity to understand the mechanisms that allow for certain cattle to respond differently to nutritional shifts.

Previous work investigated these mechanisms in the adipose tissue of mature cows [1] and discovered key metabolic and signaling pathways during CG. Another critical tissue to evaluate for transcriptional response to CG is skeletal muscle. Caton et al. [2] shows that muscle accounts for 21% of total energy; muscle and adipose combined represent 27%. The role of skeletal muscle in energy metabolism substantiates the need to understand the transcriptional response.

Keogh et al. [3] investigated the transcriptome response to CG in the muscle of bulls and found that biological processes associated with feed restriction were lipid metabolism and energy production. During realimentation, these biological processes shifted towards cellular function and organization. It is also evident that cattle differ in their ability to respond and reprogram following feed restriction. Thus, the objective of this study was to determine the transcriptional differences in skeletal muscle of cows with high or low gain during realimentation following nutrient restriction.

## Main text

The samples and phenotypes presented in this paper were collected previously [1]. Crossbred cows (n = 121) were used in the study. Angus, Hereford, and MARC III composite cows were bred by AI to Angus, Hereford, Simmental, Limousin, Charolais, Gelbvieh and Red Angus bulls. The F_1_ bulls from Angus and Hereford dams were mated to F_1_ cows from these matings to produce cross progeny. At 5 years, cows were not bred and moved to an individual feed intake facility with Calan Gates (American Calan, Northwood NH). Feed restriction and ad libitum feeding diets are described in [1]. Muscle biopsies were taken at day 105 ± 2 of the feed restriction from the left side and at day 49 ± 2 of the realimentation from the right side. The samples were immediately frozen in liquid nitrogen and stored at − 80° C.

Cows most divergent in body weight (BW) gain (12 total; n = 6 High Gain, n = 6 Low Gain) during the realimentation period were selected and skeletal muscle samples were processed. This classification was based on realimentation to identify cows with divergent BW gain during realimentation following nutrient restriction, and represent variation in CG. Cows selected for high gain had higher body weights and feed intakes than cows selected for lower gain (P < 0.0005). Cows with greater gain displayed average Gain:Feed ratio of 0.12, compared to 0.08 for cows with lesser gain (*P* < 0.01).

Total ribonucleic acid (RNA) was isolated from 50–100 mg of tissue with TriPure reagent (Roche, Indianapolis, IN), and quantified using a NanoDrop 8000 spectrophotometer (Thermo Scientific, Wilmington, DE, USA). The 260/280 measurements were > 1.8 for all samples. Quality of total RNA was assessed on an Agilent 2100 Bioanalyzer and RNA integrity number (RIN) values were ≥ 7 for all samples.

For microarray, 250 ng of total RNA was used with the Bovine 1.1ST array strips on the Affymetrix GeneAtlas System (Santa Clara, CA, USA). Transformed data were analyzed using a repeated measures model where gain class was fitted as a fixed effect and restricted and ad libitum samples as time points. Gain class was tested on the animal error term while time and its interaction with gain class was tested on the residual. Genes were considered differentially expressed when a Bonferroni adjusted *P* of < 0.05 was obtained. Raw data files were deposited in the NCBI gene expression omnibus (GEO) as series record GSE94777.

A total of 1,007 genes were differentially expressed at a nominal *P*-value < 0.05 between cows with high and low gain. However, none were significant after Bonferroni correction. After Bonferroni correction, the comparison of feed restriction and realimentation time points produced 567 differentially expressed genes (DEG; P_Bonferroni_ < 0.05; Additional file 1: Table S1). Of these, 408 genes were down-regulated and 159 were genes up-regulated during feed restriction. A heat map illustrating the 30 most up-regulated and 30 most down-regulated genes is presented in Additional file 2: Figure S1. The interaction (gain × time) analysis produced no DEG after Bonferroni correction. These data reflect a more prominent response, in terms of the numbers of DEG passing correction for multiple testing in skeletal muscle across treatment group (feed restriction versus ad libitum) rather than phenotype differences (high and low gain). Feed restriction and realimentation result in two different physiological states expressed by changes in weight gain and energy utilization [4]. Differentiation between high and low BW gain during realimentation is likely controlled by many genes with small effects, and in combination with small sample size, fewer DEG with large effects may be expected.

Validation using real-time quantitative polymerase chain reaction (RT-qPCR) was performed on five of the DEG (*INHBE*, *GNB3*, *TRHR*, *AP1M1* and *UCN*) from the analysis comparing feed restriction to realimentation (Additional file 3: Table S2). PrimePCR (Bio-Rad, Hercules, CA, USA) arrays for each target gene and *GAPDH* (housekeeping gene) were used. Real-time PCR was performed in triplicate for all samples and genes on a Bio-Rad CFX384 (BioRad) instrument using SsoAdvance SYBR Green master mix (Bio-Rad). The RT-qPCR reaction was performed according to the manufacturer’s instructions. Relative transcript abundance of each target gene was calculated using the 2^−ΔΔCt^ method [5] with the reference gene and a pooled sample. The correlation between microarray data and RT-qPCR 2ddCt values were > 0.6 for 4 of the 5 genes tested.

**Fig. 1:**
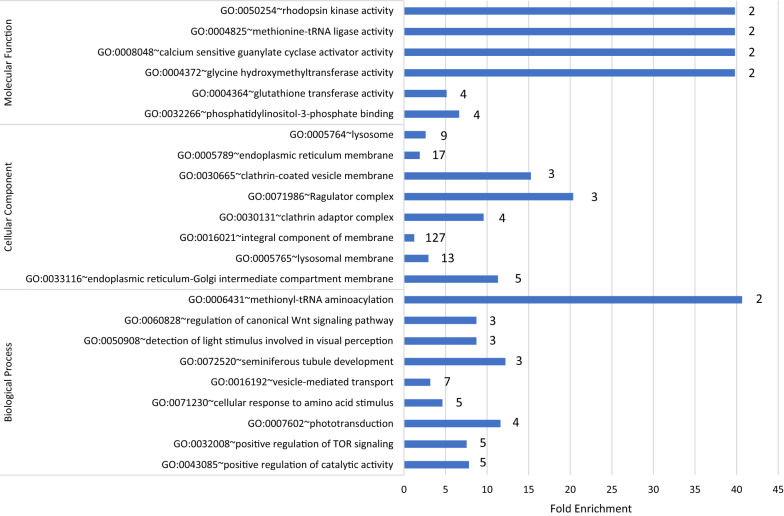
Histogram of GO classification (*P* ≤ 0.05) of differentially expressed genes in cattle fed ad libitum after feed restriction. Results are presented as biological processes, cellular components, and molecular functions. The X-axis indicates the fold enrichment of the genes identified in each category. The number of genes represented in each GO term is provided at the end of each of the bars

Functional annotation of the 567 DEG identified in the feed restriction and realimentation analysis was performed in the database for annotation, visualization, and integrated discovery (DAVID) v6.8 ([6]; Table 1; Fig. 1). Down-regulated genes were enriched for lysosome (*P* < 0.05), with trends for aminoacyl-tRNA biosynthesis, glutathione metabolism, and renin-angiotensin system pathways (*P* < 0.1). Up-regulated genes were enriched for phototransduction (*P* < 0.05). All genes identified for the lysosome pathway were expressed in higher abundance during realimentation. Lysosomes are responsible for digesting macromolecules including proteins and organelles [7]. The amino acid products of lysosome digestion are recycled for synthesis of new proteins. The upregulation of several genes in the lysosome pathway during realimentation suggests an increase in protein turnover. In support of this, roughly 7.5% of the genes identified in this study have a role in protein catalytic activity, indicating a dramatic shift in cellular response to an increase in nutrient availability.

**Table 1:** Pathways identified by Database for Annotation, Visualization and Integrated Discovery (DAVID) and Ingenuity Pathway Analysis (IPA) containing genes over-represented of cows during feed restriction and ad libitum periods

Program	Term^a^	# Genes^b^	*P*	Genes^c^
DAVID	Lysosome	10	0.0008	*CD63, NAGPA, AP1M1, AP1M2, CTSA, ENTPD4, GALC, GGA1, M6PR, MCOLN1*
	Aminoacyl-tRNA biosynthesis	4	0.06	*MARS, MARS2, SARS2, VARS*
	Glutathione metabolism	3	0.09	*GSTM2, GSTM3, SRM*
	Renin-angiotensin system	3	0.09	*ACE, ACE3, CTSA*
	Phototransduction	3	0.1	*GRK1, CNGB1, RCVRN*
	**Phototransduction**^**d**^	**3**	**0.008**	***GRK7, GUCA1A, GUCA1B***

^a^Term representing the shared functional annotation or canonical pathway of the differentially expressed genes that were over-represented in the list

^b^Number of DEG identified within the biological process

^c^Official gene symbol of the genes identified as differentially expressed for each term

^d^Term in bold represents up-regulated genes from cows during feed restriction compared to ad libitum periods. Plain text illustrates genes down-regulated during feed restriction compared to ad libitum

The DEG *MARS, MARS2,* and *VARS*, involved in aminoacyl-tRNA biosynthesis were also detected in [3]. The direction of expression was the same for both studies with an increase in transcript abundance of these genes during realimentation. These genes may be of particular interest as biological markers of CG because they have been identified in two different populations and both sexes of beef cattle [3].

Genes involved in glutathione metabolism (Table 1), an indicator of cellular stress, were upregulated during realimentation. The expression of the glutathione s-transferase (GST) genes, involved in detoxification of oxidative stress products, was higher in mature cows during realimentation. Increased expression of GST genes during CG has been reported in other studies [3, 8, 9]. Studies in mice and humans have shown that increases in caloric intake produce increases in mitochondrial production of hydrogen peroxide in muscle cells [10, 11]. Moreover, mitochondrial dysfunction and oxidative phosphorylation pathways were identified in the adipose tissue of these same cows supporting that the animals are responding to oxidative stress because of increased feed intake.

**Fig. 2:**
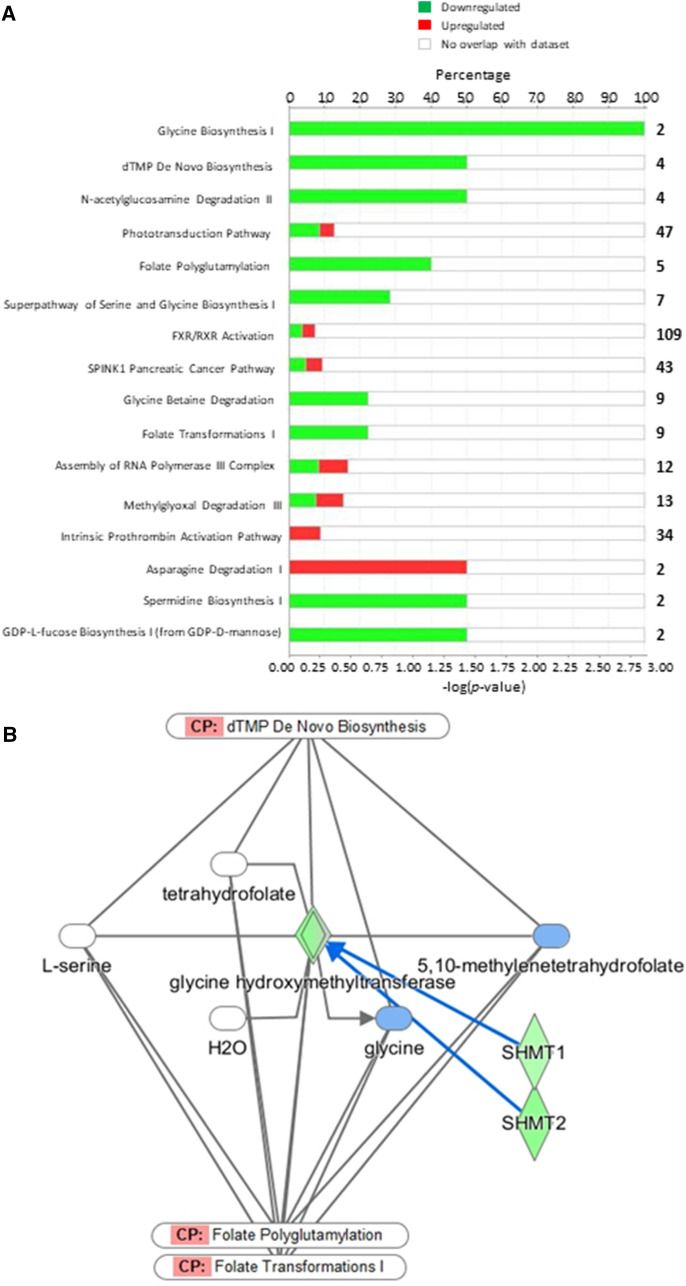
Canonical pathways identified with Ingenuity pathway analysis using the list of 567 differentially expressed genes (P_adjusted_ < 0.05). **A** illustrates the percentage of genes that were up- or down-regulated in each canonical pathway. **B** specifically illustrates the dTMP de novo biosynthesis, folate polyglutamation and folate transformations I pathways and the involvement of differentially expressed genes SHMT1 and SHMT2

The list of DEG (n = 567) between restriction and realimentation was evaluated using the PANTHER Classification System [12, 13] for over-representation in biological processes (Additional file 4: Table S3). Two biological processes were identified: cellular response to organonitrogen compound and response to acid chemical (*P* < 0.05). The genes over-represented were *SHMT1*, *SHMT2*, *LAMTOR1*, *LAMTOR4*, *LOC614531* and *RRAGA*. All were down-regulated in the feed restricted animals, and all annotated genes have functions in amino acid synthesis or cellular responses to amino acid availability. The two *LAMTOR* genes and *RRAGA* encode proteins that are crucial to the activation of the mammalian target of rapamycin complex 1 cascade to promote cell growth in response to cell signals such as nutrient and amino acid levels [14–16]. The list of DEG (n = 567) between restriction and realimentation was also analyzed with Ingenuity Pathway Analysis (IPA). Two of the canonical pathways also identified by IPA (Fig. 2A) [17], glycine biosynthesis 1 and deoxythymidine monophosphate (dTMP) de novo biosynthesis were identified via differential expression of *SHMT1* and *SHMT2* (Fig. 2B). These genes encode serine hydroxylmethyltransferase enzymes that metabolize tetrahydrofolate to 5,10-methylenetetrahydrofolate and serine to glycine [18]. Synthesis of 5,10-methylenetetrahydrofolate is a divergent point in the folate cycle with three potential outcomes, one of which is synthesis of deoxythymidine monophosphate [19]. Single-carbon biosynthesis via the folate cycle is critical for biosynthesis (proteins, polyamines, nucleotides), amino acid homeostasis, epigenetic maintenance through modification of gene expression via methylation of deoxyribonucleic acid (DNA), RNA, and histones, as well as cellular redox defense through the synthesis of glutathione mentioned previously [19–21].

We have previously evaluated the adipose from these same cows [1] and found overlap with the DEG identified in muscle. A total of 74 DEG were identified in both tissues over the course of feed restriction to realimentation. While these genes did not cluster into functional biology terms, some are of particular interest. For example, *XKR4* has been implicated in gain and feed intake in cattle [22] and *UCN* is involved in mammalian appetite and stress response [23, 24]. Genes involved in transcription or translation were also identified in both tissues (*CUX1, ETV1, EIF4EBP2, FOXJ3,* and *SOX30)*. These were all transcribed in higher abundance during realimentation in muscle. Genes with inflammatory functions were identified in the muscle tissue of cows transitioning from restriction to realimentation, including *IL18BP, IL18RAP, IL34, IL36A, INHBE,* and *SELE.* Genes *IL34, IL36A, INHBE* and *SELE* may be of particular importance, as they were detected in the adipose of these cows. These inflammatory response genes differ in function yet are important regulators of cellular response to stresses including inflammation, nutritional stress, and disease [25–31]. The identification of several common genes in both muscle and adipose tissues indicates that they share some of the same responses to CG and may be particularly important as modulators of realimentation.

The nutritional challenges implemented here reflect those experienced by cattle grazing seasonal pastures and native ranges, which represents the majority of the U.S. cow herd. The objective of this discovery study was to investigate differences in the skeletal muscle transcriptome explaining the molecular responses in BW gain in mature cows during realimentation. While some pathways associated with protein turnover and energy metabolism were identified, many of the biological processes discovered were modulators of these pathways in some capacity, even if not directly identified as belonging to those biological processes.

In summary, we identified common pathways among cows in the muscle tissue during realimentation, which included protein turnover, tRNA synthesis, and glutathione metabolism. These appear to be pathways that are critical for CG of cows during realimentation and provide insight into the underlying biological mechanisms. This study is the first to evaluate high versus low gaining mature cows for biological differences in the muscle tissue during feed restriction and realimentation. Further studies on larger groups of animals are necessary to better evaluate these genes as potential biological markers for CG.

## Limitations

A limitation of this study was the small sampling size (n = 12). There were 121 cows in this study and we selected those most divergent in BW gain during realimentation. Obtaining clear differences between animals with high versus low gain phenotype while balancing the cost to perform the microarray experiments were critical factors for our sample size. Future aims include validation of some of the most highly and lowly expressed genes detected in this study in samples from cows biopsied in different years.

## Supplementary Information

**Additional file 1: Table S1.** Statistical analysis data of the expression of genes in the muscle tissue of mature cows for high versus low gain, feed restriction versus ad libitum feed time points, and the interaction between gain and time.

**Additional file 2: Fig. S1.** Heatmap illustrating the changes in expression for the 30 most up-regulated and the 30 most down-regulated genes between the feed restriction diet and ad libitum feed.

**Additional file 3: Table S3.** Comparison of microarray data with RT-qPCR data for five genes identified as differentially expressed in the muscle tissue of cows (n = 12) fed ad libitum after feed restriction.

**Additional file 4: Table S4.** PANTHER biological processes identified as over-represented in the muscle tissue of mature cows between feed restriction and ad libitum periods.

## Data Availability

The datasets generated and/or analysed during the current study are available in the NCBI gene expression omnibus (GEO) as series record GSE94777 at https://www.ncbi.nlm.nih.gov/geo/query/acc.cgi?acc=GSE94777.

## Abbreviations

AI: Artificial insemination
BW: Body weight
CG: Compensatory gain
DAVID: Database for annotation, visualization, and integrated discovery
DEG: Differentially expressed genes
DNA: Deoxyribonucleic acid
GEO: Gene expression omnibus
GO: Gene ontology
GST: Glutathione s-transferase
IPA: Ingenuity pathway analysis
RNA: Ribonucleic acid
RIN: RNA integrity number
RT-qPCR: Real time quantitative polymerase chain reaction
