# Supplementary figures and images for: Transcriptome profiles of the skeletal muscle of mature cows during feed restriction and realimentation
Source: BMC Res Notes. 2021 Sep 16;14:361. doi: 10.1186/s13104-021-05757-8 (PMC8447676; doi:10.1186/s13104-021-05757-8)

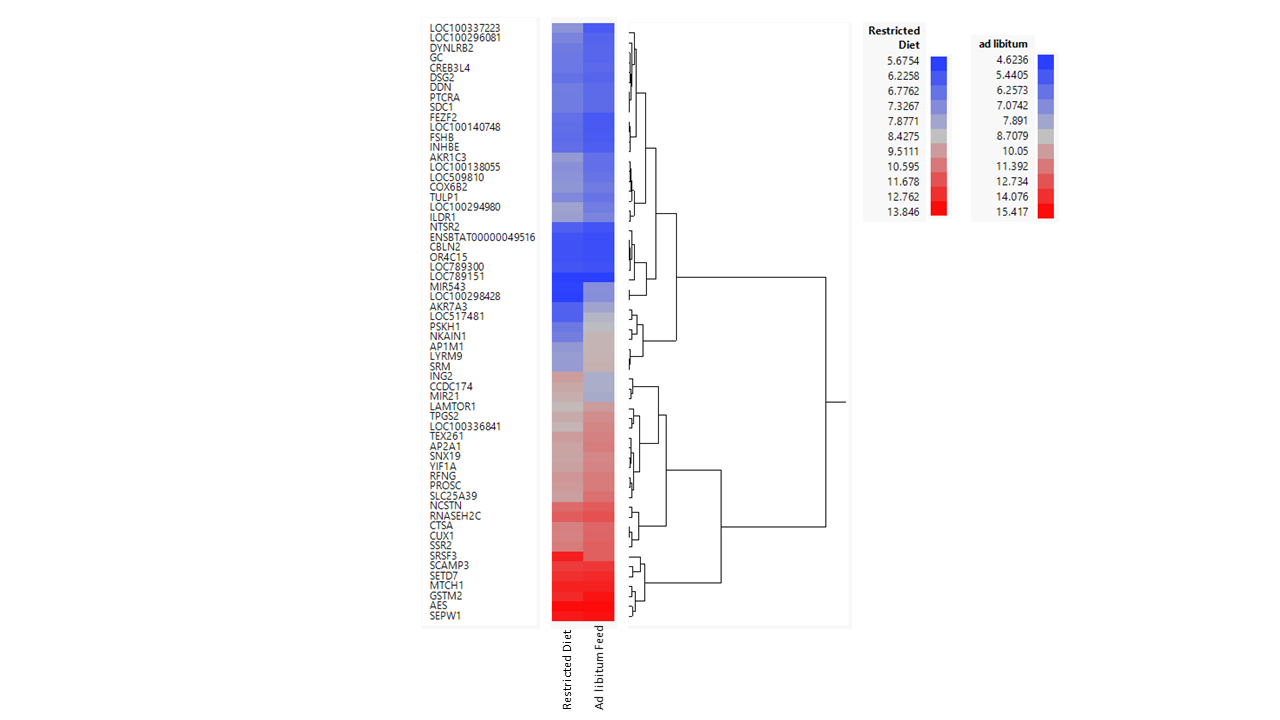

Supplement: Supplementary file 2 — Additional file 2: Fig. S1. Heatmap illustrating the changes in expression for the 30 most up-regulated and the 30 most down-regulated genes between the feed restriction diet and ad libitum feed. [file 13104_2021_5757_MOESM2_ESM.tif]
